# Water, sanitation, and hygiene insecurity and psychological distress in South Sudan

**DOI:** 10.1007/s00127-025-02934-z

**Published:** 2025-06-02

**Authors:** Rochelle L. Frounfelker, Gabrielle M. String

**Affiliations:** 1 Department of Population Health, College of Health, Lehigh University, Bethlehem, PA USA; 2 Department of Civil and Environmental Engineering P.C. Rossin College of Engineering and Applied Sciences, Lehigh University, Bethlehem, PA USA

**Keywords:** WASH insecurity, South Sudan, Mental health, War-affected populations, Concept mapping

## Abstract

**Purpose:**

There is a growing body of research on the relationship between water, sanitation, and hygiene (WASH) insecurity and mental health. The goal of this research was to understand the relationship between WASH insecurity and psychological wellbeing in one community in Juba, South Sudan, using a participatory approach with community members. We aimed to: (1) explore the kind of WASH insecurity experienced in South Sudan; and (2) hear how community members understood the relationship between these insecurities and their psychological wellbeing.

**Methods:**

A total of 56 adults from the community of Rejah, South Sudan, participated in this study. We used concept mapping to develop a conceptual framework for how community members viewed the relationship between WASH insecurity and psychological wellbeing. Participants engaged in three rounds of data collection that included brainstorming ideas, sorting ideas into meaningful groups, and providing feedback.

**Results:**

Men generated 33 statements related to WASH insecurity and psychological wellbeing, and women generated 34. Men’s ideas grouped into 5 clusters, with one focused on positive impacts; women’s ideas grouped into 6 clusters, with two focused on fears related to gender-based violence and other dangers. Together, men and women articulated a wide variety of negative impacts of WASH insecurity on psychological wellbeing, including sadness, shame, stress, worry, fear, being afraid, “thinking too much”, and low self-esteem.

**Conclusion:**

More focus is needed on the psychosocial impact of WASH insecurity among individuals in conflict and post-conflict settings. Addressing these daily stressors may have a positive impact on mental distress.

## Introduction

Water, sanitation and hygiene (WASH) insecurity is a major public health issue, particularly in low-resource settings such as developing countries and humanitarian contexts. There is a well-established body of research on the relationship between WASH insecurity and physical health [1–3], but much more limited investigation into the relationship with mental health. There is growing evidence for an association between WASH insecurity and psychological distress [4–7], which has in turn led to a call to develop and evaluate WASH interventions with a focus on how they may impact non-communicable diseases such as mental disorders [8].

Empirical research on WASH and mental health has conceptualized and measured “mental health” in a variety of ways. Some studies have assessed participants for symptoms of depression and anxiety; others evaluate general psychological distress; a few have focused on somatic symptoms and general quality of life [4, 9]. Specific to countries in Sub-Saharan Africa, water insecurity has been associated with general psychological and emotional distress [10–12] as well as symptoms of depression and anxiety [13–17]. In one study, access to handwashing facilities was associated with self-reported happiness [18]; in another, access to improved sanitation was associated with symptoms of depression [17].

Despite this growing body of empirical research, there are considerable gaps in our understanding of WASH insecurity and mental health. First, there is a need for more holistic investigations of the relationship between these factors that simultaneously evaluate different dimensions of WASH insecurity (water insecurity, handwashing facilities, and sanitation). Importantly, dynamics of psychosocial wellbeing and WASH may be context specific, calling for a need for local, regional, and country-specific analyses in relationship to this phenomenon.

This growing area of research has relevance for practitioners and researchers who focus on mental health in conflict and post-conflict settings. Among war-affected populations, it is recognized that mental health is impacted by both war-related traumas and current daily stressors, including basic necessities such as food, water, and medicine [19, 20]. Identifying and distinguishing pathways between these factors and mental health is important, as it can determine appropriate interventions for war-affected populations. In particular, there is a need to first address daily stressors before providing specialized clinical services [19]. While there is some WASH and mental health research among conflict-affected populations and populations in complex crisis [21–25] more is needed to elucidate the intricacies of this relationship.

### WASH and mental health in South Sudan

The Republic of South Sudan is one war-affected country with significant mental health and WASH challenges. Since independence in 2011, the country has seen ongoing subnational violence, two outbreaks of Civil War, and climate and economic shocks. South Sudan ranked 160 of 163 countries in the 2023 Global Peace Index, indicating significant internal conflict and low peace levels, and was the least peaceful country in the region [26]. Similarly, in 2024 the country was ranked by the World Bank as a conflict country due to the ongoing conflict as measured by conflict deaths and a rapid deterioration of the security situation [27].

Exposure to high rates of violence and insecurity has resulted in high levels of mental distress in the population. Prevalence of post-traumatic stress disorder (PTSD) is as high as 41% in the general population [28], generalized anxiety disorder at 5.5% [29], and major depression among 15.9% of the population [30]. Unsurprisingly, a decade of conflict in the young country has also impacted the WASH sector. Water and sanitation insecurity increased from 2010 to 2020, with 41% of the population accessing basic water supply services, 16% basic sanitation infrastructure, with 60% practicing open defecation, and 22% accessing soap and water for handwashing at home [31].

To date, there has been limited research on the relationship between WASH insecurity and mental health in the country. In one community survey, participants identified high levels of needs related to access to drinking water (81.1%), toilets (76.6%), and keeping clean (33.5%) [32]. Researchers found a relationship between number of needs of daily living and psychological distress [32]. In another study carried out in the country’s capital, Juba, individuals who reported a lack of food and/or water had poorer mental health than those who did not express these concerns [33]. Importantly, challenges of daily living were associated with poor mental health, and not direct exposure to war [33].

#### Present study

The goal of this research was to understand the relationship between WASH insecurity and psychological wellbeing in one community in Juba, South Sudan, using a participatory approach with community members. We used concept mapping for this work because of the importance of utilizing qualitative methods in research on this topic. Qualitative methods are particularly valuable in exploratory work, generating understanding of a phenomenon (in this case WASH and mental health), and gaining in-depth and rich information on the phenomenon from a local perspective, without imposing pre-existing assumptions [34]. We aimed to: (1) explore the kind of WASH insecurity experienced in South Sudan; and (2) hear how community members understood the relationship between these insecurities and their psychological wellbeing. Additionally, we aimed to identify gender differences in the experience of WASH insecurity and psychological wellbeing.

## Methods

### Study design and setting

Concept mapping is a participatory mixed methods research methodology that results in the development of a conceptual framework for how a group views a topic [35]. It is a structured and iterative process that engages the same participants over three sessions of data collection. This study was carried out in Juba, South Sudan in collaboration between Lehigh University, an international NGO (Oxfam), and a local NGO (Support for Peace and Education Development Programme). The protocol was carried out in accordance with the ethical standards laid down in the 1964 Declaration of Helsinki and approved by both the Lehigh University Institutional Review Board and South Sudan’s Relief and Rehabilitation Commission Ethics Board via the Director General for Planning, Training and Research.

## Participants

Participants were residents of the Juba, South Sudan area payam of Rejaf. Inclusion criteria included being aged 18 and over and able to consent to participate. Prior to conducting any research activities, study research assistants (RAs) provided an overview of study activities and obtained verbal informed consent from participants. The informed consent process and data collection activities were conducted in Juba Arabic and translated into English. A total of 25 men and 31 women participated in the study (see Table 1).

**Table 1:** Sociodemographic characteristics of study participants (*N* = 56)

	Women *N* (%)		Men *N* (%)		Total *N* (%)	
*Age*						
18–30 years	10	(32)	8	(32)	18	(32)
31–40 years	14	(45)	7	(28)	21	(38)
41 years and older	7	(23)	10	(40)	17	(30)
*Highest education level*						
None	21	(68)	5	(20)	26	(46)
Less than primary	9	(29)	3	(12)	12	(21)
Completed primary	0	(0)	10	(40)	10	(18)
Some secondary	1	(3)	2	(8)	3	(5)
Completed secondary	0	(0)	4	(16)	4	(7)
Above secondary	0	(0)	1	(4)	1	(2)
*Employment status*						
Employed with contract	1	(3)	0	(0)	1	(2)
Casual laborer	1	(3)	6	(24)	7	(13)
Self-employed (own business, vendor)	4	(13)	4	(16)	8	(14)
Unemployed	25	(81)	14	(56)	39	(70)
Missing	0	(0)	1	(4)	1	(2)
*Marital status*						
Never married	3	(10)	7	(28)	10	(18)
Married	26	(84)	17	(68)	43	(77)
Divorced/separated/widowed	2	(6)	1	(4)	3	(5)
*Parental status*						
No children	0	(0)	7	(28)	7	(13)
Children aged < 5 or under residing with you	6	(19)	4	(16)	10	(18)
Children aged 6–17 residing with you	11	(35)	7	(28)	18	(32)
Children aged < 5 AND aged 6–17 residing with you	5	(16)	2	(8)	7	(13)
Children aged 18 and over	8	(26)	5	(20)	13	(23)
*Religion*						
Muslim	0	(0)	1	(4)	1	(2)
Christian	31	(100)	24	(96)	55	(98)
*Residential status*						
Internally displaced person	0	(0)	0	(0)	0	(0)
Host community	31	(100)	22	(88)	53	(95)
Returnee	0	(0)	3	(12)	3	(5)
Refugee	0	(0)	0	(0)	0	(0)
	**Mean**	**SD**	**Mean**	**SD**	**Mean**	**SD**
*Years lived in community*	24.9	(14.9)	30.1	(16.7)	27.2	(15.8)

## Data collection

Concept mapping data collection took place over three rounds over a two-week period. RAs (one male and one female) were trained by the authors to conduct each round of data collection and facilitate focus group discussions. RAs were bilingual in English and Juba Arabic.

*Round 1.* The goal of the brainstorming session is to generate ideas, comments, statements, and observations [35]. The brainstorming sessions were facilitated separately for men and women by the study RAs. At the beginning of the session, the facilitator guided study participants as to the purpose of the group, planned activities, and general guidelines. The facilitator provided participants with working definitions of WASH and psychological wellbeing for the purposes of the study. RAs asked participants to respond to the following prompts:

1. One water issue that impacts psychological wellbeing, either positively or negatively, is….
2. One sanitation issue that impacts psychological wellbeing, either positively or negatively, is….
3. One hygiene issue that impacts psychological wellbeing, either positively or negatively, is….

Study staff wrote down all statements and ideas in English.

*Round 2.* The goal of the second round of data collection is to structure the list of previously generated statements based on perceived similarity [35]. The study team created sets of flashcards for the men and women and asked participants to work individually, one at a time, to sort the items. The study RA presented each flashcard to the participant, explaining the idea in Juba Arabic and presenting the images to represent the idea. After going through each image, participants were invited to group the items with the following instructions [35]:

1. All statements cannot be put in a single pile;
2. All statements cannot be put into their own separate piles;
3. Each statement can be placed in only one pile.

This activity generated unique pile sorts of statements for each study participant.

*Round 3.* The goal of the third-round focus group is to obtain feedback on the final cluster map. Flashcards that belonged to a cluster were taped together onto a flipchart with the cluster title in English and Juba Arabic. The study RAs presented each cluster/flipchart page separately, and asked the following questions of the group:

1) How do these ideas relate to each other?

2) How does this group of ideas relate to psychological wellbeing?

3) What would you need to address this group of items?

4) What must change to get what you need?

Focus groups were audio recorded.

### Data analysis

After the conclusion of Round 1, study personnel consolidated ideas brainstormed by each group and developed a master list of all ideas generated, separated by gender. The authors reviewed items for clarity, ratability, relevance to WASH insecurity and psychological wellbeing, and duplication. In conversation with RAs, phrasing, accuracy, and clarity of statements was reviewed. Study leads generated final lists for men and women. Because the majority of participants were illiterate, each idea was printed on a flash card and team members used images/icons to visually represent each statement.

After Round 2, the study PIs analyzed data from the pile sorting using GroupWisdom concept mapping software [36]. This analysis consists of 3 steps: (1) Creating a similarity matrix that shows the number of participants who sorted each pair of statements together in their sorts; (2) Multidimensional scaling of the similarity matrix; and (3) Hierarchical cluster analysis of the multidimensional scaling. Separate two-dimensional maps were generated for men and women. The study PIs evaluated cluster maps using criteria of: (1) interpretability and; (2) goodness of fit to the data to select the final number of clusters from the hierarchical cluster analysis. Additional exploratory analysis was conducted to assess any trends in responses based on participant age and parental status. Lastly, cluster labels to define the statements grouped together were generated with study RAs in English and translated into Juba Arabic.

After Round 3, focus groups were translated and transcribed from Juba Arabic to English. Data were analyzed in MAXQDA [37] by the authors using a deductive approach to coding and content analysis [38, 39]. For instance, researchers used codes that mapped onto each of the focus group prompt questions, including “how items are related,” “relationship to psychosocial wellbeing,” and “needs.” Researchers also used and applied codes for each distinct cluster developed by men and women, and analyzed data to identify similarities and differences between men and women.

## Results

### Round 1: brainstorming

Men generated 33 statements related to WASH insecurity and psychological well-being (see Table 2), and women generated 34 (see Table 3). Both men and women identified issues related to water that impact psychological wellbeing, including lack of clean water and difficulty getting water (particularly in the dry season). Men brought up challenges associated with flooding, including contamination of water supplies and preventing access to schools and health care facilities. Although men did raise concerns about gangs when accessing water at the river, women brought up this and several other fears regarding going to the river to use water, such as rape, attacks from wild animals, evil spirits, and children being vulnerable to attacks. Specific to sanitation, men mentioned the importance of latrines and dangers of accessing latrines at night. Women spoke more about sanitation issues including, reasons for, and shame in, open defecation, dangers in open defecation, and sanitation and hygiene challenges related to menstruation. Men shared ideas related to both personal and household hygiene practices related to psychological wellbeing. They spoke of how hygiene was associated with respect from the community and self-esteem; in contrast, not being hygienic would lead to shame, isolation, and poor physical health. For women, they spoke about the stresses associated with not being able to be hygienic, including concerns for the health of children and lack of access to products (see Tables 2 and 3).

**Table 2:** Men’s brainstorming statements

Statements
M1. Drinking clean water makes me happy
M2. Polluted water from the river makes me sad
M3. Walking far to the river decreases family time and time to do productive things
M4. When water is hard to pump it is frustrating
M5. Accessing water from the river is unsafe because of gangs/armed gangs (makes you feel worried/scared)
M6. Flooded rivers cause fear of being swept away
M7. Flooding affects access to hospitals and schools for children
M8. Poor hygiene makes you uncomfortable being in public with others and impacts self-esteem
M9. Poor hygiene is stressful because the community might think you are mad/have a mental illness
M10. Long distance to access water is stressful
M11. Having a clean home earns respect from the community
M12. When a man does not maintain good hygiene he smells and it causes marital problems
M13. Having a clean home makes the owner happy and reduces stress
M14. Accessing latrines at night is scary because of snakes
M15. Open defecation makes you an outcast in the community
M16. Poor sanitation and hygiene lead to sickness
M17. A clean home prevents disease and keeps wild animals away
M18. A clean home brings good health
M19. A dirty home brings wild animals and disease
M20. Not being hygienic leads to damaging your brain and becoming mad
M21. Good hygiene prevents sickness and germs
M22. Flooding brings diseases in the water
M23. Bathing every day makes you feel healthy
M24. Dirty water leads to diarrhea/other diseases
M25. Lack of water leads to dehydration
M26. Having a dirty home makes you not feel good
M27. A dirty home will smell bad
M28. Bathing in dirty water leads to rashes and itching
M29. A water shortage leads to drinking contaminated water
M30. Everyone has a responsibility to make a hygienic home
M31. When there is access to water you can do domestic tasks (wash, cook, grow food)
M32. When you have an unclean home people will not visit you (isolation might make you feel crazy)
M33. When you are bathed and wearing clean clothes you will be respected

**Table 3:** Women’s brainstorming statements

Statements
W1. Open defecation can lead to snake attacks
W2. Open defecate at night to avoid being attacked/killed by animals and gangs (causes fear)
W3. Children may be attacked at night going to the latrines (causes fear)
W4. Evil spirits can catch you at the river at night and make you made (causes worry)
W5. Evil spirits can take adults and children at the river at night
W6. A crocodile can attack when you bathe in the river (causes worry)
W7. Getting water from boreholes at night can lead to rape by men (causes stress)
W8. Defecating in the open can lead to rape
W9. Guardians are traumatized if their children are raped going to the river
W10. Getting water from the river can lead to being attacked/raped by men/gangs (causes worry, shame and isolation)
W11. Being seen bathing in the river is shameful
W12. Urinating blood after showering in river
W13. Smelling in public is shameful
W14. Guardians are stressed if children get rashes from not bathing or bathing in dirty water
W15. Sharp grass can poke your bottom when defecating in the open (causes fear)
W16. Defecating in the open leads to shame
W17. Not having a latrine makes me sad
W18. No latrines mean you walk long distances and worry if you have diarrhea (causes shame)
W19. No latrines for disposing fecal waste
W20. Latrines lead to good hygiene
W21. Having water means you can clean yourself in the latrine
W22. Hygiene kits prevent disease
W23. When there is water it improves hygiene and sanitation
W24. Collecting water with other women is a safe place to discuss life
W25. Not having money to buy WaterGuard, detergent, or soap causes stress
W26. Lack of water when you have visitors causes stress
W27. Not having enough water makes you think too much
W28. Stagnant water causes disease
W29. Children and adults can drown in the river when fetching water
W30. Even if there is no food, you can drink clean water and feel healthy
W31. A water source used for all activities (bathing, washing) can become contaminated
W32. In the dry season you walk far and lose time to get water
W33. Water can be polluted with waste and insects when it rains (causes stress)
W34. Lack of sanitary pads during menstruation leads to getting blood on your clothes (causes shame)

### Round 2: sorting

**Fig. 1:**
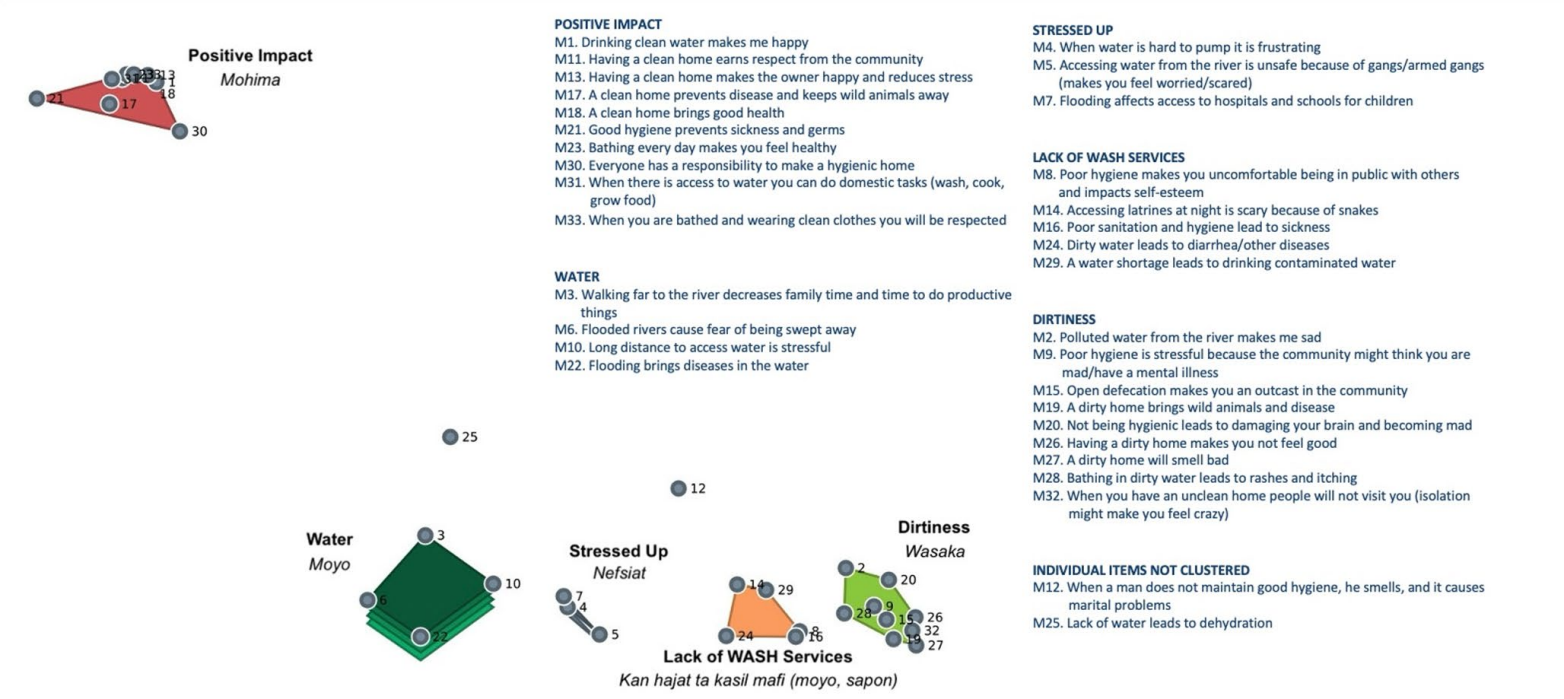
Men’s concept mapping of WASH and mental health

Among the men, individuals grouped the 33 items into between 2 and 7 clusters (mean = 4.6, SD = 2.02). The men’s map included 5 clusters and two individual statements (see Fig. 1). Items were grouped into clusters labeled “Positive Impact (*Mohima*),” “Water (*Moyo*),” “Stressed Up (*Nefsiat*),” “Lack of WASH Services (*Kan hajat ta kasil mafi [moyo*,* sapon]*),” and “Dirtiness *(Wasaka)*.” For the men, not surprisingly, all items that clustered under WASH issues that positively impact psychological wellbeing were located geographically on the map at a far distance to all the other items. The clusters *Water* and *Stressed Up* both contained statements related to water, with the distinction that *Stressed Up* statements focused more specifically on access issues that lead to stress or fear. The cluster *Lack of WASH Services* included topics related to the downstream psychological consequences of physical health problems that result from lack of proper water, sanitation, and hygiene resources in the community. The cluster *Dirtiness* centered on issues related to practicing good hygiene in the home and maintaining personal cleanliness.

**Fig. 2:**
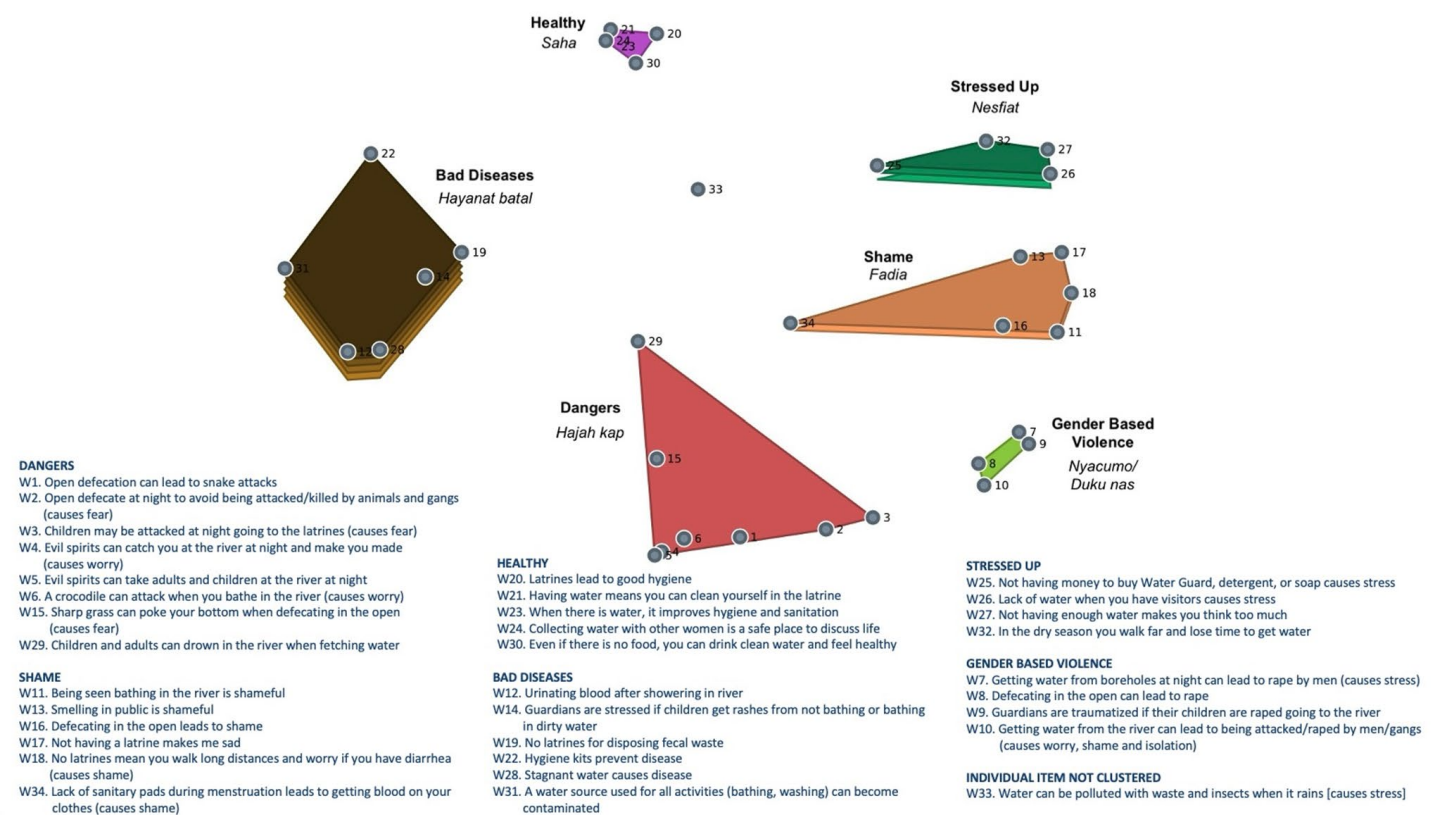
Women’s concept map of WASH and mental health

Among the women, individuals grouped the 34 statements into between 3 and 8 clusters (mean = 5, SD = 1.22). The women’s map included 6 clusters and one individual statement (Fig. 2). Items grouped into clusters labeled “Bad Diseases *(Hayanat batal)*,” “Healthy *(Saha*),” “Stressed Up *(Nesfiat*),” Shame *(Fadia)*,” Gender-Based Violence *(Nyacumo/Dukun as)*,” and “Dangers *(Hajah kap*).” As with the men, almost all ideas that related to positive impacts of WASH clustered together, although not as geographically distant from other clusters as with the men’s map. Most statements in the cluster *Bad Diseases* focused on negative consequences of the lack of WASH services. As with men, the women’s *Stressed Up* statements focused on stressors related to access to water, with additional concerns for not being able to purchase sanitation and hygiene products. The *Shame* cluster was in closest proximity to *Stressed Up*, and focused on feeling shame related to not having access to sanitation and not being able to practice good hygiene. Women identified a separate cluster, *Gender-based Violence*, focused entirely on fear of sexual assault and rape that they and their children endured because of WASH insecurity. Finally, *Dangers* contained ideas related to other threats to physical well-being when seeking water and/or accessing sanitation services, including attacks from animals and evil spirits.

### Round 3: discussion

When discussing each cluster, men had interesting observations about the relationships between items grouped together. One person identified the challenges and stress of not being able to take care of things typically considered issues of personal responsibility. When speaking about the cluster *Dirtiness*, he noted,

“…when you are not clean, your thinking capacity lowers because everyone will look at you like you are not physically fit. This cleanness here, it’s supposed to be your own responsibility as a person. But if you don’t have the things to get what you need, it’s a problem. You will never be clean because you don’t have the soap to wash clothes.”

Men connected access to WASH services with overall happiness and life satisfaction. One person commented,

“In my wellbeing, if I’m always clean, I’ll be mentally fit. You will always be happy because your clothes are clean, drinking clean water, they are cooking food for you, you are always bathing, you are stress free. You feel very light, you feel like you don’t have any issues affecting you.”

Men identified a variety of ways that WASH insecurity was related to their mental health. Several commented that personal lack of hygiene and dirtiness was stressful because they might be ostracized by the community. Speaking of the cluster *Lack of WASH services* one man stated, “not washing my clothes, not washing my hands, people look at me in the community like someone who is mentally ill and unstable. And that is related to making me stressed because I am very dirty.” This stigma had consequences on social relationships and may lead to isolation. One individual explained when discussing the *Stressed Up* cluster, “People in the community will isolate you after seeing that you are mad mentally…If someone is stressed, he or she will not feel happy in the community, he will be mentally confused.” Another person noted that this stress could ultimately have an impact on overall community functioning, as he stated, “People in the community will not have relationships between themselves because everyone lives in a stressful way.” This was in stark contrast to a comment made about the cluster *Positive Impact*: “I gain confidence in the community because everybody in my place is known and respected.”

The women’s group discussed at length negative outcomes of WASH insecurity on psychological well-being, expressing how they experience stress, shame, mental thinking, and trauma. Many women discussed how statements in the *Bad Diseases* cluster caused a lot of thinking and stress, as diseases were related to a lack of clean water, latrines, and hygiene materials. One woman explained,

“How it affects me. If your home is always dirty and you don’t even have money for buying soap, for washing your clothes, for bathing. And also, it brings some diseases like malaria and makes you to vomit. If it happens to you or your child like that, at times it will bring you like someone mad, because you are thinking a lot, so it affects your mind.”

When discussing the *Shame* cluster, women frequently discussed the many challenges associated with menstruation due to a lack of access to products. Shame during menstruation was associated with blood being visible because of not having sanitary pads, or odor being noticeable because of the lack of soap for bathing, as demonstrated by,

“For example, there is plenty of water, but there is no soap. But if you have period you bathe, and the smell is there, and later you go in the middle of people. When you say to them, ‘what is wrong?’ And they move from you, and you will feel bad, and you just stand up and walk away, which brings shame and stress.”

Stress was the most frequently used word by the women to explain the challenges of WASH insecurity. In the *Stressed Up* cluster in particular, one woman described how insecurity caused overlapping stresses, “The long-distance to go fetch water will really stress you a lot. Many times your kids are home alone, you need something to eat, but at the same time you have to go far to collect water. It will stress you, the woman.” Importantly, some woman in the group noted that the experience of WASH insecurity is not uniform across the community, and some experience more stress and difficulties than others. This was exemplified by, “For us who are already old, we are the ones who are affected more than these young ladies.” Women described the fear and resultant stress that came from thinking about attacks that could happen to themselves or their children after running into gangs, animals, or evil spirits while collecting water, defecating in the bush, bathing in the river, or walking to the latrines. The statements in the *Gender-based Violence* cluster were discussed by women as being related to feelings of fear, stress, and shame. They explained how walking at night to get water or access latrines made them vulnerable to attacks, “At night hours, if you go to the borehole and you will be raped from there it affects you internally and it will bring stress and shame, and even fear of the night hours.”

Not all discussions focused on negative psychological wellbeing, and women noted how having WASH was related to reduced stress, health, and strengthened community. For instance, despite the challenges of collecting water from distant sources, while discussing the *Healthy* cluster, women also stated how important it was to be in community with other women during water collection:

“You go there with your stress from your home, and you are stressed, or you are annoyed…You will come to the group and you will realize that this other woman, her problems are the worst, and you will console yourself from there at that meeting place…It will reduce your stress. You have talked everything inside your heart and you will come right from the borehole to your home without any stress.”

Participants were asked what they needed to address WASH insecurity issues that affected psychological wellbeing. During the discussions, participants recommended solutions for WASH insecurity in their community, ranging from securing soap, menstrual hygiene products, insect nets, scythes, and gumboots to building infrastructure like latrines, boreholes, and pumped water distribution systems. Often the community expressed addressing water issues is the key to sanitation and hygiene challenges in the community.

## Discussion

This study found multiple connections between WASH insecurity and psychological wellbeing in the community. In addition to highlighting negative consequences of insecurity, individuals discussed how WASH security can promote individual and community psychological wellbeing. Men’s ideas grouped into 5 clusters, with one focused on positive impacts; women’s ideas grouped into 6 clusters, with two focused on fears related to gender-based violence and other dangers. Notably, the clusters of *Shame* (Fadia) and *Stressed Up* (Nesfiat) mapped onto culturally relevant mental health problems in Juba Arabic identified in previous qualitative research conducted in Juba, South Sudan [40]. Symptoms of *Stressed Up* (Nesfiat) include thinking too much, feeling worried, and being stressed because there is no solution for a problem [40]. Symptoms of *Shame* (Fadia), include feeling guilty, worried that people will laugh at you, fear of talking openly and looking into someone’s eyes, being quiet, and feeling discouraged [40]. Together, men and women articulated a wide variety of negative impacts of WASH insecurity on psychological wellbeing, including sadness, shame, stress, worry, fear, being afraid, “thinking too much”, and low self-esteem. WASH insecurity also had implications for social relationships, and could lead to isolation, lack of respect from community members, and loneliness. Interestingly, men discussed the stigma of lack of cleanliness and good hygiene, with perceptions that a person had a mental illness (“being mad”). Conversely, women described “going mad” as a result of being attacked by evil spirits while bathing or collecting water at the river.

An important outcome of this study is reporting gender stratified data on psychological wellbeing and WASH insecurity. There are few studies that have documented the experience of men in this space. Water insecurity was related to 11 of the 23 statements made by men not grouped in the *Positive Impact* cluster and a smaller portion (13 of the 29 statements) made by women not grouped in the *Healthy* cluster. This contrasts a previous study in Bolivia that found significantly fewer men overall reported negative psychological wellbeing outcomes of water insecurity compared to women [41]. In this study, men noted that open defecation makes you an outcast in the community, which is similar to a systematic review of sanitation insecurity that found men experienced shame when practicing open defecation [6]. More research with men and boys about their experience with WASH insecurity and psychological outcomes is needed to develop WASH programs that address these needs.

Many more studies have reported on the mental health and psychological wellbeing of women experiencing WASH insecurity [42, 43]. Similar psychosocial consequences of water insecurity (worry, shame, anger, and fear) were noted by women in Kenya [44], who also discussed the stress of not having clean water for visitors, risk of gender-based violence, and fear of animal attacks. Among a study of refugees in Uganda, adolescent girls and young women had increased risk of sexual and gender-based violence due to water scarcity and distant water infrastructure [24]. Additionally, women globally have been documented in a systematic review on sanitation insecurity as experiencing fear and stress from animal attacks, gender-based violence, and shame when defecating in the open [6].

### Limitations

There are several limitations to this study. First, our results are focused on one community in Juba, South Sudan, and may not be generalizable to other populations in South Sudan or other conflict-affected settings. However, our methods provide a rich description of WASH insecurity and mental health that is culturally relevant, an important step in understanding context-specific needs and identifying potential interventions to address both mental health and WASH concerns. The high illiteracy rate of the population meant that concept mapping activities had to be modified to accommodate the participants, which could have reduced variation in brainstorming and for the women during pile sorting as they wanted to help give input to each other’s answers. However, concept mapping has been conducted by others using icons and pictures [45]. A final limitation of this work is that we do not have quantitative data to measure prevalence of disease or symptoms of depression in this population; future work should include a mixed methods approach to further explore the epidemiology of WASH and mental health.

### Implications

Despite these limitations, the study has important implications. Previous studies have found that “disgust” and “status” were positively associated with returnees and internally-displaced people living in village settings to participate in handwashing activities [22]. Herein, this study demonstrates that there may be a gendered component to similar outcomes, as men were concerned with “Dirtiness” in the study more than woman, highlighting the need for WASH implementers and mental health practitioners to be aware of these nuances.

Overall, participants self-generated solutions to address WASH insecurity in their community, including securing household items and constructing infrastructure; with gendered differences, likely based on WASH and psychological wellbeing experiences, driving the varied responses. Securing smaller household items may help to alleviate short-term stressors and shame experienced by both men and women to improve dignity and agency. Recommending WASH infrastructure to meet the intertwined insecurity challenge of a risk of gender-based violence among a conflict-affected population is common [24]. Many of the stressors, shames, and worries expressed by community members of Rejaf are likely to continue unless infrastructure is implemented to address long-term WASH insecurity issues. This is important for WASH implementers to know when considering impacts of interventions beyond physical health, as well as for health service providers to be aware of when evaluating the mental health of community members.

## Conclusion

Overall, this study highlights that water scarcity, poor water quality, open defecation, and lack of hygiene items contribute in a myriad of ways to the short and long-term mental stressors of a war-affected population. Understanding how these daily stressors compound with other effects on the mental health and psychosocial well-being of communities affected by conflict is important as the effects of climate change and migration increase globally.

## Data Availability

The datasets used and analyzed during the current study are available from the corresponding author on reasonable request.
